# Methods for Enrichment and Assignment of N-Acetylglucosamine Modification Sites

**DOI:** 10.1074/mcp.R120.002206

**Published:** 2021-02-09

**Authors:** Jason C. Maynard, Robert J. Chalkley

**Affiliations:** Department of Pharmaceutical Chemistry, University of California San Francisco, San Francisco, CA, USA

**Keywords:** Electron transfer dissociation, glycosylation, o-glcnacylation, mass spectrometry, PTM analysis, PTM enrichment, proteomic database searching, BEMAD, Beta-elimination followed by Michael addition of DTT, CID, collision-induced dissociation, ETD, electron transfer dissociation, EThcD, electron transfer dissociation combined with collision induced dissociation, HexNAc, N-acetylhexosamine, OGA, O-GlcNAcase, O-GlcNAc, O-linked N-acetylglucosamine, OGT, O-GlcNAc transferase, LWAC, lectin weak affinity chromatography, WGA, wheat germ agglutinin

## Abstract

O-GlcNAcylation, the addition of a single N-acetylglucosamine residue to serine and threonine residues of cytoplasmic, nuclear, or mitochondrial proteins, is a widespread regulatory posttranslational modification. It is involved in the response to nutritional status and stress, and its dysregulation is associated with diseases ranging from Alzheimer’s to diabetes. Although the modification was first detected over 35 years ago, research into the function of O-GlcNAcylation has accelerated dramatically in the last 10 years owing to the development of new enrichment and mass spectrometry techniques that facilitate its analysis. This article summarizes methods for O-GlcNAc enrichment, key mass spectrometry instrumentation advancements, particularly those that allow modification site localization, and software tools that allow analysis of data from O-GlcNAc-modified peptides.

## Highlights

• O-GlcNAc enrichment methods
• Mass spectrometry methods for O-GlcNAc analysis and site identification
• Software tools to assist in O-GlcNAc data analysis
• New dataset identifies over 700 sites of O-GlcNAc modification in human monocytes

## Graphical Abstract

**Figure:**
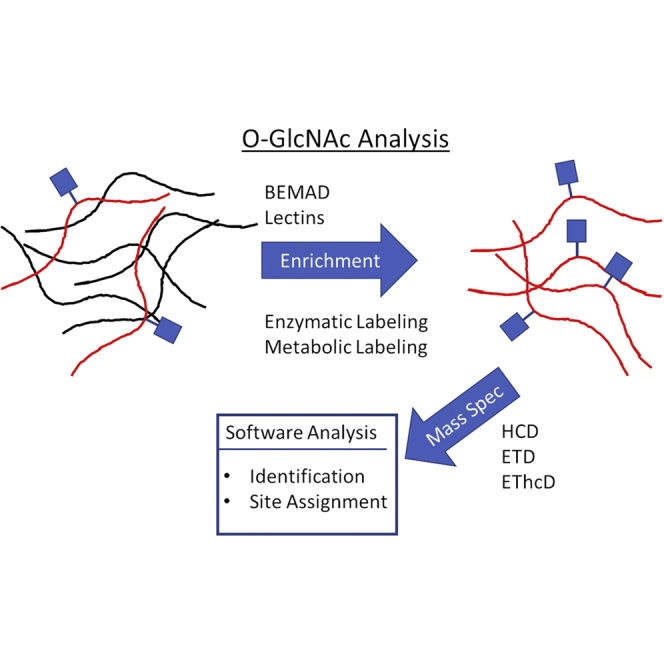

## In Brief

This review article summarizes methods for O-GlcNAc enrichment and different mass spectrometric approaches for acquiring data on modified peptides and describes software strategies for analyzing data, including the challenges of reliably identifying modification sites and differentiating between other potential HexNAc modifications. It then presents a new dataset to exemplify what is currently achievable.

## O-GlcNAcylation is a Widespread Regulatory Modification

The cell uses several posttranslational modifications to transiently regulate a protein’s activity. One of the most important and widespread modifications is the addition of a single β-N-acetylglucosamine (GlcNAc) sugar residue to serine and threonine residues of nuclear, cytoplasmic, and mitochondrial proteins. This is a regulatory modification whose addition and removal are each catalyzed by a single enzyme, uridine diphospho-N-acetylglucosamine:polypeptide β-N-acetylglucosaminyltransferase (OGT) and O-GlcNAcase (OGA), respectively. The donor molecule is UDP-GlcNAc, which is the product of the hexosamine synthetic pathway and is thought to be a sensor for glucose levels (1). As a result, O-GlcNAcylation is broadly a response to nutritional status and stress (2). It regulates gene transcription, protein translation, and has a complex interplay with phosphorylation (3), in some cases competing for the same site of modification. O-GlcNAcylation is found in practically all multicellular organisms. In humans it is dysregulated in neuropathological disorders such as Alzheimer’s disease and metabolic disorders such as diabetes and is elevated in most cancers. For a more comprehensive description of O-GlcNAc’s many roles see this recent review article (4).

## Early Methods for O-GlcNAc Detection

The O-GlcNAc modification was serendipitously discovered over 35 years ago through glycan radiolabeling experiments of lymphocytes (5). The authors expected to label terminal N-acetylglucosamine residues of cell surface glycans using a galactosyltransferase but also found considerable labeling in the nucleus and cytoplasm. This radiolabeling approach (mostly using tritiated galactose) was the dominant method of detection for the first 20 years of studying the O-GlcNAc modification. Early attempts were made to use mass spectrometry to detect modified peptides; however, researchers were unable to determine modification sites in these peptides using the then available tandem mass spectrometry instrumentation. As alternatives, radiolabeling and Edman sequencing were used to determine modification sites (6, 7). Owing to instrument developments discussed later in this review, mass spectrometry has now become the dominant method for O-GlcNAc site determination.

This article will only focus on methods that allow modification site determination, but in many cases there are related approaches that can be used to visualize modified proteins through blotting or fluorescent labeling (8, 9, 10, 11).

## Modification Enrichment Is Required

Most tandem mass spectrometry data are acquired using a process known as data-dependent acquisition, where the instrument automatically selects the most intense ions for fragmentation analysis. Modified peptides are generally not among the most abundant species in a sample because modifications are typically substoichiometric, so unmodified peptides will dominate. In a large-scale reanalysis of published proteomic datasets to look for GlcNAc-modified peptides only 126 O-GlcNAc-modified peptides were found among just under 14 million spectra analyzed (12). Hence, for comprehensive analysis of O-GlcNAcylation an enrichment step is required to move modified peptides to among the most abundant in the sample such that during data-dependent acquisition they are automatically selected for fragmentation analysis.

## Lectin Enrichment

Nature produces many sugar-binding proteins that are referred to collectively as lectins. One well-studied lectin is wheat germ agglutinin (WGA), which is used by the plant in its defense system. WGA’s highest affinity is to terminal GlcNAc residues, and it also has lower affinity to sialic acids (13). WGA forms dimers with two binding sites within each subunit. These act in a co-operative manner, such that WGA can bind tightly to glycans with multiple terminal GlcNAcs. Several researchers have tried to use WGA to create a method for O-GlcNAcylated peptide enrichment but found that the affinity was too low to perform bind-and-elute enrichments. However, effective chromatographic methods based on retardation of modified peptides when passed through a column of WGA, referred to as lectin weak affinity chromatography (LWAC), have been developed. The first of these used WGA-agarose resin, but it was found that very long columns, up to 12 m, were desirable to get useful separation (14). Two significant improvements have since been made. The first was using POROS resin, which provides a higher density of WGA per unit length, allowing shorter columns to be used. The second was the realization that the separation efficiency was hampered simply by the vast excess of unmodified peptides, meaning the tail of the unmodified peptides coeluted with the modified peptides. By collecting this tail and then reloading for a second or even third round of LWAC highly enriched glycopeptide fractions (>70% modified peptides) can be produced (15). WGA-based LWAC chromatography enriches nearly all types of N- and O-glycosylation, not just O-GlcNAc. In a whole-cell lysate the O-GlcNAc-modified peptides will be a subset of the total glycopeptides in the sample. Preparing a cytosolic, or nuclear, preparation before performing WGA-LWAC will produce more highly enriched O-GlcNAc fractions, but even then, it is common to get contamination from the endoplasmic reticulum (ER), golgi, and lysosomal proteins. Hence, it is important to confirm that peptides identified as being modified by a single HexNAc are O-GlcNAc and not N-GlcNAc or O-GalNAc modified.

Succinylated WGA does not bind sialic acid residues, so should have higher specificity for O-GlcNAc over some other glycoforms. However, our attempt to use this for LWAC was not effective. This is probably because succinylated WGA has a lower affinity than WGA for GlcNAc (16) such that the interaction is not strong enough for chromatographic enrichment.

## Enzymatic Labeling

The original galactosyltransferase labeling approach has been adapted as a method for modified peptide enrichment. The galactosyltransferase was mutated to allow the addition of a galactose containing an unnatural ketone group that can be used to attach an affinity tag such as biotin (17). This tag was used for measuring changes in O-GlcNAcylation in the brain following stimulation (18). However, the large tag hampers the ability to identify modified peptides as one gets extensive tag fragmentation rather than peptide backbone. Hence, a photocleavable linker was added, such that after enrichment the biotin moiety can be removed before mass spectrometric analysis (19). This method was used to study cross talk between O-GlcNAcylation and phosphorylation during cytokinesis (20). A photocleavable tag has also been developed that incorporates isotopic labeling that allows quantitative comparison of two samples (21), and this was used to discover differences in O-GlcNAcylation in drug-resistant *versus* drug-sensitive HepG2 cells. More recently, a variation of this protocol was developed that attached an affinity tag linked *via* a disulfide bond that could be reduced for tag cleavage and removal after enrichment (22).

As described earlier, the original method for detecting O-GlcNAcylation was through the enzymatic labeling of terminal GlcNAc residues with galactose. This terminal galactose can be enriched using the lectin Ricinus Communis Agglutinin I such that labeled O-GlcNAc-modified proteins can be enriched (23). This approach has mostly been used for enrichment of modified proteins but has been demonstrated for modified peptide enrichment (24).

## Metabolic Labeling

Rather than introducing a handle for tagging by enzymatic addition of a second sugar residue it is possible to introduce a functional group directly. The O-GlcNAc transferase is able to add an azide analog of GlcNAc referred to as GlcNAz, so one can study new O-GlcNAcylation through metabolic incorporation of this azide sugar, followed by enrichment and mass spectrometric analysis (25). An alkyne equivalent can also be employed in the same way (26).

An elaborate strategy for glycopeptide analysis is Isotope-targeted glycoproteomics (IsoTaG). This approach uses metabolic labeling to introduce the affinity handle, but then adds an isotope-encoded tag containing two bromines (27). Bromine has two stable isotopes of masses 79 and 81 Da at roughly equal abundance. As a result, peptides labeled by this tag produce a unique isotope pattern that allows them to be recognized in the mass spectrometer as modified. This can be useful if one does not have an efficient enrichment strategy such that only a small percentage of peptides are modified, as it allows one to produce a list of peptide masses that are modified that can then be targeted for fragmentation analysis in a subsequent run. Using this strategy for O-GlcNAc analysis just over 2000 modified peptides corresponding to several hundred modification sites were identified among tryptic and chymotryptic digests of T cells (28).

A limitation of metabolic labeling is that one is competing with endogenous UDP-GlcNAc for incorporation, so the stoichiometry of modification is never going to be high, leading to a significant sensitivity hit. In the large-scale IsoTaG study (28), enrichment was performed at the protein level such that only one site in the protein needed to be GlcNAz-modified for enrichment. In the subsequent analysis, many of the modification sites were identified as GlcNAc rather than GlcNAz-modified, exemplifying the incorporation issue. Protein-level enrichment leads to a high percentage of unmodified peptides being present, which is why the isotope pattern introduced using IsoTaG is useful to find modified peptides.

## Immunoprecipitation

One limitation of all the enrichment methods described thus far is the lack of specificity for O-GlcNAc over other types of glycosylations containing GlcNAc. A few antibodies have been raised against O-GlcNAcylated proteins that have shown some affinity to O-GlcNAcylation in general. The best known of these are the RL2 antibody that was raised against nuclear pore complex proteins (which are heavily O-GlcNAcylated) (29) and CTD110.6 (10), which binds the heavily O-GlcNAcylated C-terminal domain of RNA Polymerase II. A set of monoclonal antibodies were produced using a designed three-component immunogen including a synthetic O-GlcNAc-modified peptide for protein-level enrichment (11) and in a subsequent study were used to identify 83 O-GlcNAc modification sites (30). However, it is clear that each of these monoclonal antibodies only recognize a subset of modified proteins.

To produce a specific pan-O-GlcNAc enrichment approach the Van Aalten group have attempted to make use of an enzymatically dead mutant of OGA (31). This version of the enzyme still binds O-GlcNAc but is unable to release the moiety. Using a GST-tagged version of their mutant OGA enzyme they could visualize modified proteins on a 1D gel with an anti-GST antibody, but it has yet to be demonstrated that this mutant OGA could be used for immunoprecipitating modified proteins or peptides.

## BEMAD

As discussed below, the highly labile nature of the glycosidic linkage in O-GlcNAc-modified peptides makes modification site assignment extremely difficult in collision-induced dissociation data. One approach to bypass the high lability of the glycosidic linkage is to chemically derivatize the modified site, replacing it with a more stable moiety that could also be used as an affinity handle. Strong base–catalyzed beta-elimination of O-linked glycopeptides is a commonly used approach for releasing glycans for subsequent study. This reaction produces an unsaturated carbonyl on the formerly modified serine or threonine, which can then be modified by a nucleophilic attack reaction. This chemistry has been used to create an approach referred to as Beta Elimination followed by Michael Addition of DTT (BEMAD). The DTT derivatization introduces a stable modification with a free thiol that can be used for enrichment using thiol chromatography. This allows for the identification of formerly O-GlcNAcylated sites (32). A complication of this approach is that the same beta elimination reaction occurs (albeit at a slower rate) for other O-linked modifications such as phosphorylation and alkylated cysteines, and it can even occur at low levels on unmodified serines and threonines. Hence, it is important to have additional information to confirm that the former modification was O-GlcNAc. One demonstrated approach to address this is to split a sample and treat half with a glycosidase. BEMAD with normal DTT (d0) or deuterated DTT (d6) is performed on each half. Formerly glycosylated peptides will produce a single peak, whereas formerly phosphorylated or unmodified serines will produce pairs of peaks at equal intensity (33). BEMAD has also been used for modification site localization after using a chemoenzymatic glycopeptide enrichment protocol (12).

Table 1 presents a summary of the discussed enrichment methods and their different strengths, weaknesses, and biases.

**Table 1:** Summary of O-GlcNAc enrichment methods

Enrichment	Binding affinity	Specificity	Enriches all O-GlcNAc	Comments	References
WGA Lectin	Weak	Enriches most glycopeptides	Yes	Probably the highest sensitivity of the methods owing to not requiring a labeling step before enrichment	(14, 15, 39, 40, 42, 45)
BEMAD	Medium	Enriches most O-linked modifications	Yes	As original modification is replaced, it is important to have independent verification that it was O-GlcNAc	(32, 33)
Enzymatic Tagging	Strong	Enriches terminal GlcNAc-containing glycopeptides	Yes		(17, 19, 20, 22, 41)
Metabolic Tagging	Strong	Enriches all types of GlcNAc-containing glycopeptides	Subset of newly modified	Lower sensitivity owing to competing UDP-GlcNAc levels leading to substoichiometric incorporation	(25, 28, 56)
Immunoprecipitation	Weak	Enriches subset of O-GlcNAc	Each enriches a subset	Will only enrich some modified peptides	(10, 11, 30)

## Mass Spectrometry for O-GlcNAc Analysis

**Fig. 1:**
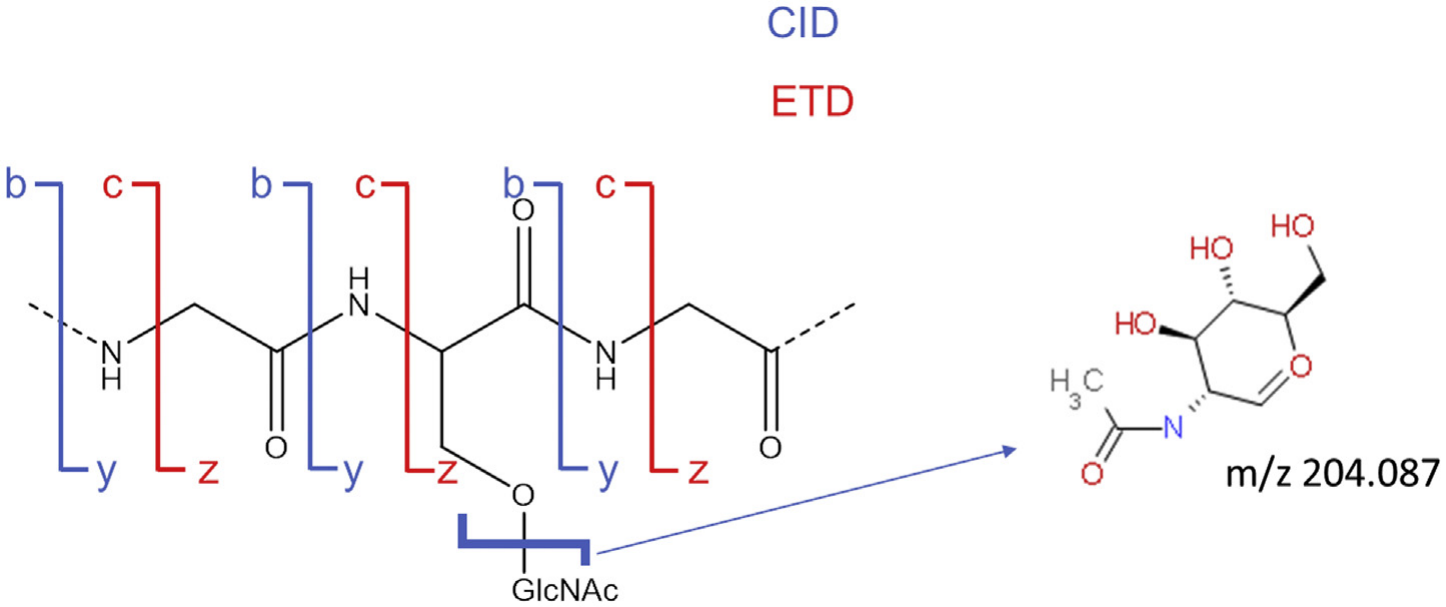
**Major bond cleavages in collision-induced dissociation (CID) and electron transfer dissociation (ETD) of glycopeptides.** The primary cleavage in CID fragmentation of HexNAc-modified peptides is of the glycosidic bond resulting in a 204.087 Da oxonium ion.

Most methods for peptide fragmentation in the mass spectrometer involve introducing internal energy by colliding the peptides with neutral gas molecules. These collision-induced dissociation (CID) methods cause the weakest bonds in the molecule to break. Unfortunately, the weakest bonds in a glycopeptide are glycosidic linkages. When an O-GlcNAcylated peptide is subjected to CID the primary fragmentation is cleavage of the bond between the GlcNAc and the side chain of the attached amino acid. This leads to fragment ions that correspond to the oxonium ion of the GlcNAc at m/z 204.087 and the unmodified peptide (34). Fragmentation in an ion trap only puts energy into the precursor ion, so an ion trap fragmentation spectrum essentially only contains these ions. In a quadrupole collision cell such as in a quadrupole-TOF or quadrupole-Orbitrap instrument fragment ions can undergo further collisions, so fragments that are the product of peptide backbone cleavages are also observed (b and y ions, see Fig. 1), allowing the peptide sequence to be determined, but as these are mostly deglycosylated fragment ions there is generally not enough information to determine the site of glycosylation.

One approach to try to circumvent this problem was to derivatize the modification site. Using a base-catalyzed beta-elimination reaction the formerly modified site is observed with a mass that is a water smaller than an unmodified residue. However, as mentioned above, this reaction can occur for other O-linked modifications, so measuring the peptide before and after the elimination reaction was used to determine the modification sites (34).

A series of improvements in mass spectrometry instrumentation has since transformed the ability to identify O-GlcNAcylation sites directly. The first of these improvements was the commercial availability of quadrupole-TOF instruments. Compared with triple quadrupole or ion trap instruments, quadrupole-TOF instruments vastly improved signal-to-noise, such that low intensity O-glycosylated fragment ions could be detected for the first time (35). This allowed for the first identification of O-GlcNAc modification sites directly using tandem mass spectrometry (36).

The next instrument breakthroughs were the development and commercial availability of new fragmentation methods. Rather than breaking the weakest bonds in the molecule and producing b and y ions, these new methods break molecular bonds at sites of electron capture to produce c and z ions (see Fig. 1). The first of these methods was electron capture dissociation (37), which allowed the detection of 12 sites of O-GlcNAcylation from a mouse postsynaptic density preparation (14). It was then shown that electron transfer from an anion was more efficient than using a beam of electrons (38). Electron transfer dissociation (ETD) has since become the method of choice for O-GlcNAc peptide identification and site localization. The first demonstration of using ETD for O-GlcNAc analysis identified seven modification sites in neurons (18). By combining ETD analysis with lectin enrichment, 58 sites of O-GlcNAcylation were identified from mouse postsynaptic density (39). This roughly doubled the number of known modification sites at the time. Shortly thereafter, this list of sites was dwarfed by a more extensive study of O-GlcNAcylation and phosphorylation that reported over 1750 O-GlcNAc sites (15). Since then, a handful of studies have been published identifying hundreds of sites at a time (20, 28, 40, 41, 42). Some of these studies have made use of a combination of HCD and ETD data, where the HCD data are used to identify a precursor as being HexNAc-modified based on the formation of the HexNAc oxonium ion at m/z 204.087; then matched ETD data are used for the identification of the peptide and modification site (30, 39). Acquisition software can be told to only perform ETD fragmentation if a given precursor forms the HexNAc oxonium ion (or fragments of this ion), which is useful if the sample contains many unmodified peptides, for example, if enrichment was performed at the protein rather than at the peptide level (30). As discussed below, newer studies of this type will likely make use of a combination of ETD and CID fragmentation referred to as EThcD (43). Some peptides fragment better by one or other type of activation, so by combining fragmentation types a higher percentage of precursors provide an informative spectrum, although the fragments formed by ETD are generally necessary for modification site localization.

## Software for O-GlcNAc Analysis

**Fig. 2:**
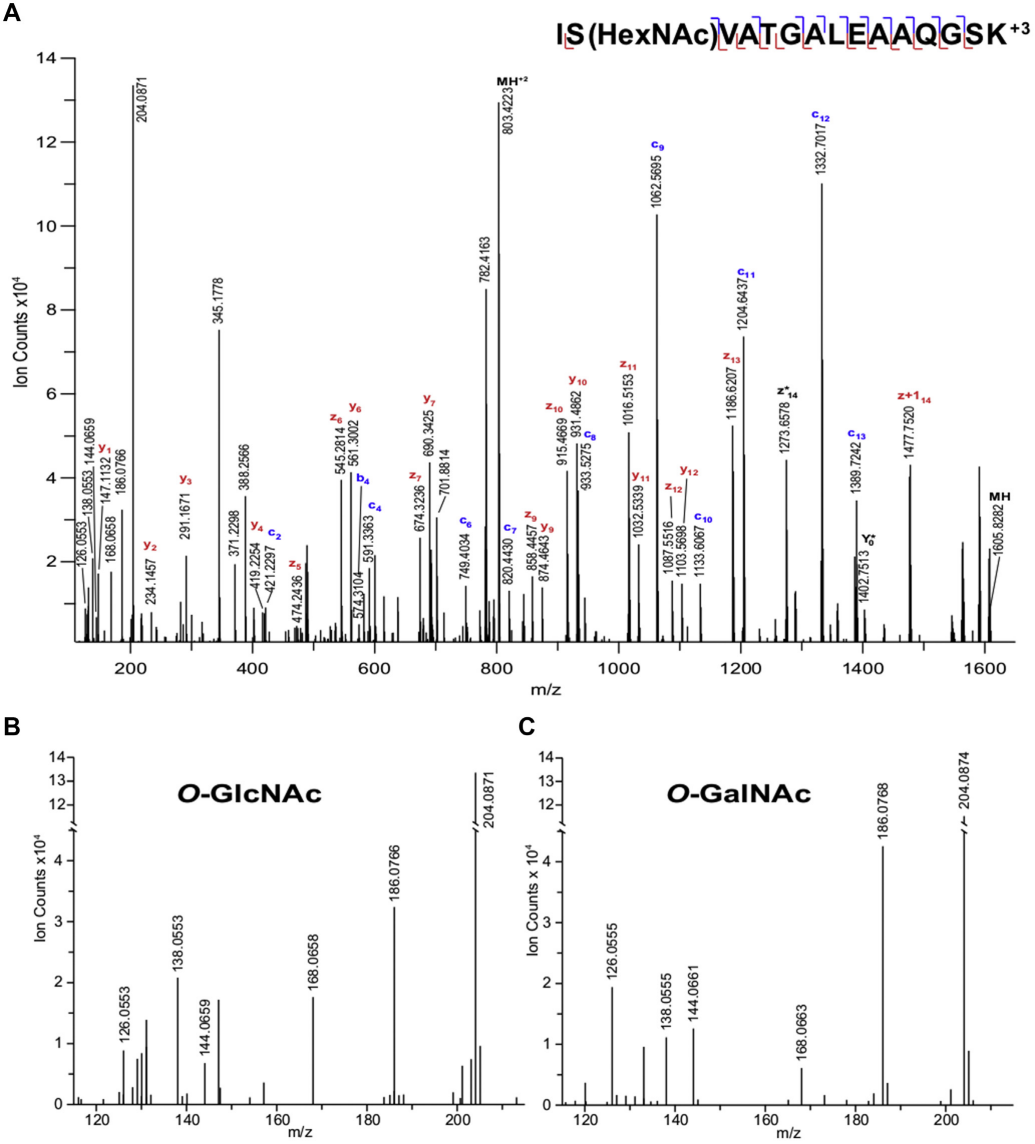
**O-GlcNAc- and O-GalNAc-modified peptides can be differentiated in EThcD spectra.***A*, annotated EThcD spectrum of an O-GlcNAc-modified peptide from Host Cell Factor 1. The fragments c_2_ and z + 1_14_ localize the HexNAc moiety to serine 1150. Ions with a glycan loss are represented with an ∗. *B*, low mass region of the spectrum in *A*. The intensity of m/z 138 is much higher than that of m/z 144, indicating the modification is O-GlcNAc. *C*, low mass region of spectrum shown in supplemental Fig. S1. Ions at m/z 138 and m/z 144 are of similar intensity, suggesting the peptide is O-GalNAc modified.

The diagnostic loss of the GlcNAc modification in CID can be used to identify spectra of modified peptides. The m/z 204.087 GlcNAc oxonium ion is specific to glycopeptides. Unfortunately, this ion is produced from other types of glycosylation in addition to O-GlcNAcylation: N-linked glycosylation produces the same GlcNAc-derived fragment and extracellular O-GalNAc-linked glycosylation produces the same mass ion. However, there are methods to differentiate O-GlcNAcylated peptide spectra from these other types of glycosylation. Extended glycans produce many other glycan oxonium ions, most notably an ion corresponding to HexNAcHex at mass 366.139 Da, so the presence of larger glycan ions can be used to exclude spectra from O-GlcNAc assignment. As stated, the GalNAc oxonium ion has the same mass as the GlcNAc oxonium ion. However, the two isomers both further fragment into a series of ions (m/z 126.055, 138.055, 144.065, 168.066, and 186.076) and the relative intensity of these fragment ions can be used to differentiate between GlcNAc and GalNAc (44, 45) (and see Fig. 2). Specifically, when O-GlcNAc further fragments the m/z 138 ion is always significantly more intense than the m/z 144 ion, whereas O-GalNAc produces these fragments at a similar intensity. EThcD fragmentation spectra can both identify modification sites and differentiate between HexNAc isomers.

The O Score software was written to identify spectra as potential glycopeptides based on the presence of the m/z 204.087 ion and its fragments (46). More powerful software along the same theme is MS-Filter in Protein Prospector (47). When presented with a peak list from a mass spectrometry run MS-Filter can be used to create new peak list files that either contain or do not contain peaks corresponding to the ion(s) of interest. With multiple rounds of filtering, peak list files can be generated that include all spectra that contain the m/z 204.087 peak while removing those that also contain the m/z 366.139 peak (and potentially other glycan oxonium ions) to filter out N- and extended O-GalNAc-linked glycopeptide spectra.

Most database search engines can be adapted to analyze O-GlcNAcylation. The primary complication is that, in CID-type fragmentation, the software needs account for the precursor ion to be shifted by the mass of the modification (+203.080 Da), but assume all fragments are unmodified, whereas in ETD it should assume the modification remains on the fragment ions. As such, several search engines have been used for O-GlcNAc peptide identification including Protein Prospector (39), Mascot (12), and OMSSA (22), and Byonic has been used for the analysis of IsoTaG data (28). An important aspect of any software for O-GlcNAc analysis is that it evaluates modification site localization. In the case of Protein Prospector this is in-built (48); for some other software it may be necessary to run the results through separate tools to evaluate which sites can be reliably identified (49). EThcD data analysis of O-GlcNAc-modified peptides is more complicated, as fragments from ETD activation are expected to retain modifications, but those from HCD activation will not. In-house analysis of EThcD data using Protein Prospector allowing for both loss and retention of the modification on fragments has indicated that modification site localization becomes complicated: if you observe a fragment modified owing to ETD, but an equivalent fragment unmodified owing to CID, this can create ambiguity. In our experience assuming that all fragments are modified in EThcD data produces more reliable modified peptide identifications and site localizations, even if it means a few fragment ions are not explained.

## O-GlcNAcylation Site Prediction

The human genome encodes over 500 kinases for the addition of a phosphate group to proteins. The fact that there is only a single O-GlcNAc transferase, OGT (albeit with three splice variants), that is responsible for thousands of modifications begs the question as to how it can have sequence specificity. Analyses of identified modification sites have shown a strong enrichment for a proline two or three amino acids prior to the modification site, and serines and threonines preferentially in all other surrounding positions. It is also common to find several residues modified in close proximity, so multiply-modified peptides are frequently detected. The proline residue introduces a kink in the protein chain, which may make regions of the protein more accessible to an enzyme for modification, but OGT probably does not formally have any further sequence preference. OGT is often thought of as corresponding to a catalytic subunit that is part of a larger protein complex, the other components of which are responsible for targeting to specific proteins for modification.

A database of known O-GlcNAcylation sites, dbOGAP, was published about 10 years ago (50), but this has since disappeared. dbOGAP attempted to predict O-GlcNAcylation sites, and other tools have also been developed for modification site prediction (51), but these all have high error rates, so should be used with caution.

## How a Recent Global O-GlcNAcylation Study Performs

Some of the largest O-GlcNAc datasets created thus far have been from our group using lectin weak affinity chromatography with WGA (15, 40). These studies employed ETciD for fragmentation analysis. As an example of the amount of information that can be achieved in global O-GlcNAcylation studies using the newer EThcD technique we present an experiment where O-GlcNAcylation was studied in human monocyte cells.

We started with 20 mg of THP1 whole-cell lysate to enrich glycopeptides. In our experience, the amount of starting material required for LWAC varies from sample to sample, although in general, the higher the amount of material the more the O-GlcNAcylated peptides are recovered. We typically try to start with at least 10 mg of protein from a whole-cell lysate, although we have had excellent results with as little as 5 mg starting material (unpublished results). The tryptic peptides from the whole-cell lysate were split into 10 injections for glycopeptide enrichment using LWAC. The separate injections are to try to minimize overloading of the column, which reduces the resolution of separation of the glycosylated peptides from the unmodified majority. The tails of each of these runs were collected together using an in-line C18 column and eluted in one fraction. After this first enrichment step it is typical that about 5% of peptides are glycosylated. Two subsequent rounds of LWAC enrichment were then performed. The resulting enriched glycopeptide sample was fractionated offline by high-pH reverse-phase chromatography to generate 50 fractions. Pairs of these fractions were combined (fraction 1 + fraction 26, 2 + 27, etc.) to generate 25 fractions that were run on an Orbitrap Fusion Lumos using EThcD fragmentation. Based on the percent of obtained spectra that contained a HexNAc oxonium ion at m/z 204.087, 41% of precursors were glycosylated. This is at the low end for enrichment using LWAC. We typically achieve 40% to 70% modified peptides depending on the sample.

We used MS-Filter to generate a set of HexNAc oxonium ion containing peak lists. These were searched against the human SwissProt database allowing for nearly 300 glycan compositions on N, S, and T. This resulted in the identification of over 8000 unique glycopeptides, the vast majority of which contained extended N- and O-linked sugars. These extended glycan IDs are useful to help distinguish N-GlcNAc and O-GalNAc modifications from O-GlcNAc modifications. We also used a known subcellular localization if available and the intensity ratio of the HexNAc oxonium ion fragments discussed above to confirm HexNAc modifications as O-GlcNAc. After manually verifying the O-GlcNAc-modified peptides, we identified just over 1800 unique O-GlcNAc-modified peptides from 420 proteins. We were also able to identify over 700 sites of O-GlcNAcylation from this dataset by thresholding at a 5% false localization rate threshold at the spectrum level using the SLIP scoring in Protein Prospector (48), which typically leads to around 1% incorrect site assignments at the dataset level. These O-GlcNAcylated peptides and the unique modification sites localized are summarized in supplemental Tables S1 and S2, and annotated spectra can be viewed through a web browser in MS-Viewer submission gi6ztunb9r (52).

Global analysis of O-GlcNAcylated peptides reveals information about what proteins and pathways in a given system are regulated by O-GlcNAc modification. Various analyses, such as interaction networks in STRING (53) or functional analyses with PANTHER (54) or DAVID (55), can be performed on these lists of proteins to infer the role of O-GlcNAcylation in the system being studied. Supplemental Fig. S2 and supplemental Table S4 show functional interaction network analysis of the O-GlcNAc-modified proteins in our THP1 dataset created using stringApp and Cytoscape. Clear functional networks emerge including mRNA splicing, clathrin-mediated endocytosis, mitotic cell cycle, and protein transport in the nucleus and ER. The proteins found to be involved in ER protein transport are cytosolic membrane–associated proteins, not located within the ER (where one should not find any O-GlcNAc modification). High confidence site localization is of great importance to researchers attempting to study mechanistic insights into particular O-GlcNAcylated proteins.

## Conclusions

The ability to analyze O-GlcNAcylation of proteins on a large scale, as well as to reliably identify exact sites of modification has been transformed by the development of enrichment strategies and new fragmentation techniques in the mass spectrometer. Several enrichment methods have been developed, but there is no one method that can selectively enrich all O-GlcNAc-modified peptides without also enriching other types of glycosylation. Nevertheless, owing to the fast speed of modern mass spectrometers it is now possible to routinely identify many hundreds of O-GlcNAc-modified peptides in a single study, although modification site localization within peptides is still not a formality and must be assessed. ETD (or hybrid fragmentation such as EThcD) is generally necessary for modification site localization, but even using these fragmentation techniques some spectra provide ambiguous results as to the exact site of modification. Hence, use of software that can estimate false-localization rates is essential (49). Differentiating between O-GlcNAc and other single HexNAc modifications is also required. Using site localization software can help recognize N-linked GlcNAc. In addition, the presence of the consensus N-glycosylation motif (N-!P-S/T, where !P is anything other than a proline) is a strong clue (although O-GlcNAc has been found on serines and threonines in this motif). To differentiate O-GlcNAc from O-GalNAc the relative intensity of their fragment ions can be used. Knowing the protein localization also allows one to assign O-GlcNAc over other types of glycosylation. Availability of a database of previously identified O-GlcNAc proteins and sites to replace the previously useful dbOGAP would be a useful resource for the field. In the future, all these types of information for glycosylation type differentiation could be assessed automatically using software. Nevertheless, it is now possible to perform global quantitative analyses of O-GlcNAc modification in response to stress or stimulation, so an improved understanding of the many signaling pathways regulated by O-GlcNAcylation is starting to be uncovered.

## Experimental Procedures

### Sample Preparation

THP1 cells were grown in RPMI 1640 supplemented with 5% fetal calf serum, 1X penicillin/streptomycin, 1X glutamine, and 1X fungizone. Cells were pelleted, washed with PBS twice, and then sonicated in 50 mM ammonium bicarbonate containing 8M urea, 4X Phosphatase Inhibitor Cocktails II and III (Sigma-Aldrich), and 40 mM PUGNAc (Tocris Bioscience). Protein concentrations were estimated with bicinchoninic acid protein assay (ThermoFisher Scientific). The protein lysate was reduced for 1 h at room temperature with 5 mM DTT and subsequently alkylated using 10 mM iodoacetamide for 45 min at room temperature in the dark. Lysates were diluted to 2 M urea using 50 mM ammonium bicarbonate, pH 8.0, and digested overnight at room temperature with sequencing grade trypsin (ThermoFisher Scientific) at an enzyme to substrate ratio of 1:50 (w/w). Following digestion, samples were acidified using formic acid (Sigma-Aldrich), desalted using a 35 cc C18 Sep-Pak SPE cartridge (Waters), and dried to completeness using a SpeedVac concentrator (Thermo).

### Lectin Weak Affinity Chromatography

Glycopeptides were enriched as described previously (15, 45). Briefly, 20 mg of desalted tryptic peptides were resuspended in 1000 μl LWAC buffer (100 mM Tris pH 7.5, 150 mM NaCl, 2 mM MgCl2, 2 mM CaCl2, 5% acetonitrile) and 100 μl was run over a 2.0 x 250-mm POROS-WGA column at 100 μl/min under isocratic conditions with LWAC buffer and eluted with a 100-μl injection of 40 mM GlcNAc. Glycopeptides were collected inline on a C18 column (Phenomenex, Torrance, CA, USA). Enriched glycopeptides from 10 initial rounds of LWAC were eluted with 50% acetonitrile, 0.1% FA in a single 500-μl fraction, dried, and LWAC enrichment was repeated for a total of three enrichment steps.

### Offline Fractionation

Glycopeptides were separated on a 4.0 × 150-mm Gemini 5μ 110A C18 column (Phenomenex). Peptides were loaded onto the column in 20 mM NH4OCH3, pH 10, and subjected to a gradient from 2% to 9% over 2 ml then from 9% to 50% 20 mM NH4OCH3, pH 10, in 90% acetonitrile over 20 ml collecting 50 fractions.

### Mass Spectrometry Analysis

Glycopeptides were analyzed on an Orbitrap Fusion Lumos (Thermo Scientific, San Jose, CA, USA) equipped with a NanoAcquity UPLC (Waters, Milford, MA, USA). Peptides were fractionated on a 50 cm × 75 μm ID 2 μm C18 EASY-Spray column using a linear gradient from 3.5% to 30% solvent B over 185 min. Precursor ions were measured from 375 to 1500 m/z in the Orbitrap analyzer (resolution: 120,000; AGC: 4.0e5). Each precursor ion (charged 2–7+) was isolated in the quadrupole (selection window: 1.6 m/z; dynamic exclusion window: 30 s; MIPS Peptide filter enabled) and underwent EThcD fragmentation (Maximum Injection Time: 250 ms, Supplemental Activation Collision Energy: 25%) measured in the Orbitrap (resolution: 30,000; AGC; 5.0e4). The scan cycle was 3 s.

Peak lists for EThcD were extracted using Proteome Discoverer 2.2. EThcD peak lists were filtered with MS-Filter, and only spectra containing a 204.0867 m/z peak corresponding to the HexNAc oxonium ion were used for database searching. EThcD data were searched against human entries in the SwissProt protein database downloaded on July 31, 2019, concatenated with a randomized sequence for each entry (a total of 40,862 sequences searched) using Protein Prospector (v6.1.10). Cleavage specificity was set as tryptic, allowing for two missed cleavages. Carbamidomethylation of Cys was set as a constant modification. The required mass accuracy was 10 ppm for precursor ions and 30 ppm for fragment ions. Variable modifications included methionine oxidation, protein N-terminal methionine removal and/or acetylation, pyroglutamate formation from peptide N-terminal glutamine and a total of 295 different N- or O-linked glycosylation compositions, which are listed in supplemental Table S3. For N-linked glycosylations they were required to occur in the motif N-!P-S/T, where !P is any amino acid other than proline. One N-linked and up to four O-linked modifications per peptide were permitted. Unambiguous PTMs were determined using a minimum SLIP score of six, which corresponds to a 5% local false localization rate (48). Modified peptides were identified with a peptide false discovery rate of 1%. O-GlcNAc and O-GalNAc modifications were differentiated based on known protein subcellular localization and HexNAc oxonium ion fragment ratios (44).

### O-GlcNAc Network Analysis

Network analysis was performed in Cytoscape v3.7.1 with stringApp v1.4.2. The network analysis used a confidence score cutoff of 0.9 and the functional enrichment analysis used an FDR value cutoff of 0.05.

## Data Availability

Raw data have been uploaded to the MassIVE repository with the identifier MSV000085653. Annotated spectra, peak lists, and the table of results for the annotated spectra can be viewed and downloaded from MS-Viewer with the keyword gi6ztunb9r (52).

## Conflict of interest

The authors declare that they have no conflicts of interest with the contents of this article.
