# Supplementary material for: Methods for Enrichment and Assignment of N-Acetylglucosamine Modification Sites
Source: Mol Cell Proteomics. 2021 Feb 9;20:100031. doi: 10.1074/mcp.R120.002206 (PMC8724609; doi:10.1074/mcp.R120.002206)
Supplement: Supplemental Figures S1 and S2 [file mmc1.pdf]

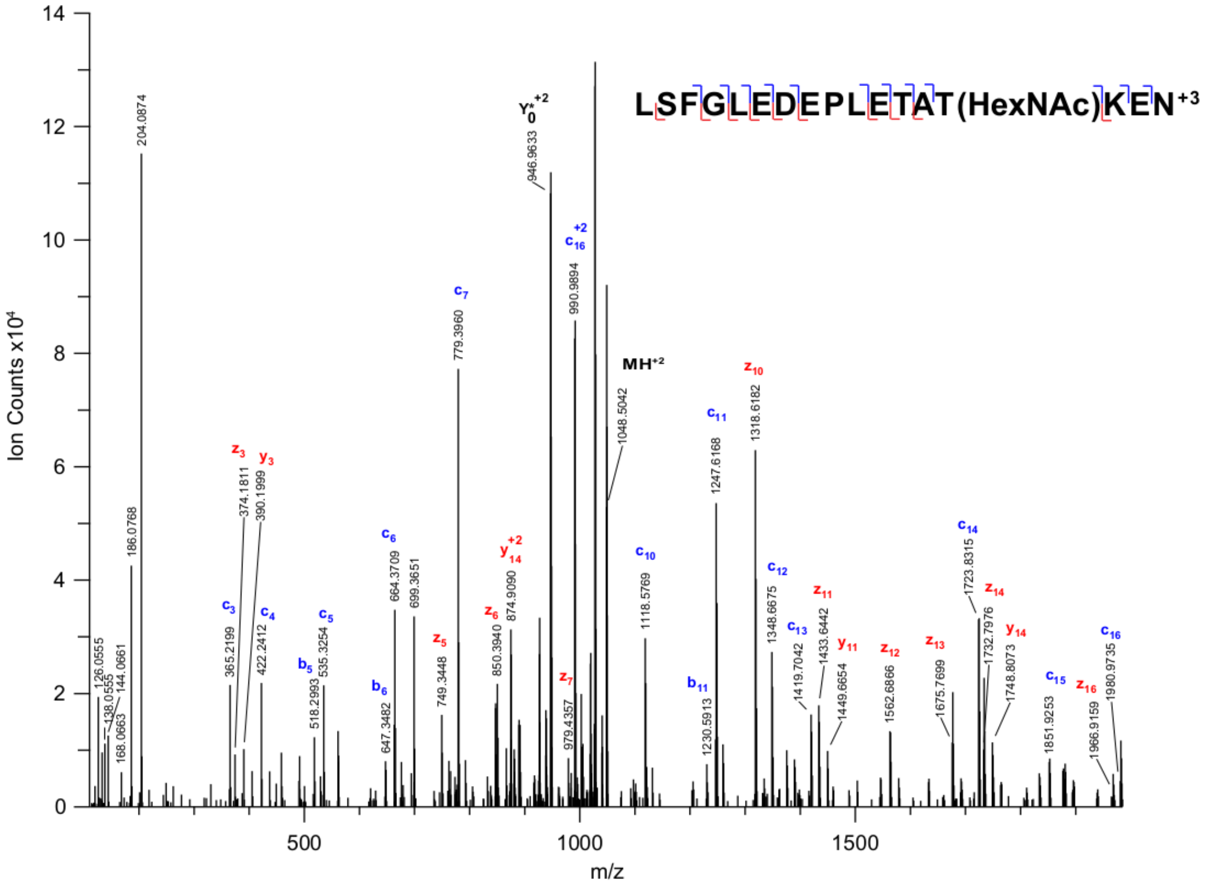

Supplementary Figure S1. Annotated EThcD spectrum of O-GalNAc modified peptide from Erlin-2. The fragments c_{14} and z_5 localize the HexNAc moiety to threonine 336. Ions with a glycan loss are represented with an *.

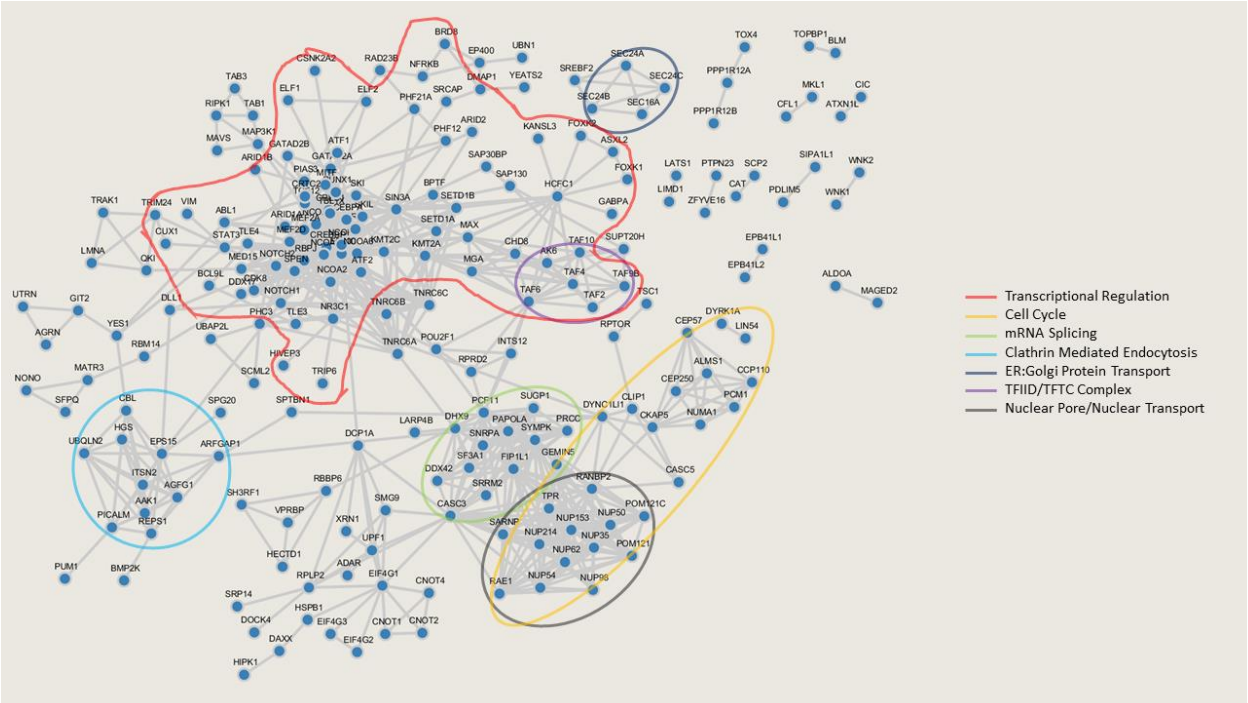

Supplementary Figure S2. O-GlcNAc modified protein network analysis using Cytoscape and stringApp. Proteins with at least one connection are shown.
